# A Novel *ZmLG1* Allele as a Genetic Resource for Breeding Compact Plant Architecture in Maize

**DOI:** 10.3390/genes17091075

**Published:** 2026-09-06

**Authors:** Yue Feng, Haimo Zhu, Shiyi Zhong, Lijing Wang, Yan Zhao, Wei Xu, Qingzhi Liu, Shengkun Liu, Baoshen Liu, Yongzhong Zhang

**Affiliations:** College of Agronomy, Shandong Agricultural University, Tai’an 271018, China

**Keywords:** maize, map-based cloning, functional markers, *ZmLG1*

## Abstract

Background: Leaf angle is a key determinant of plant architecture and yield under high-density planting. Loss of the ligule reduces leaf angle, offering a strategy for breeding compact maize. This study aims to identify the genetic basis of the liguleless mutant *jd30* and develop a functional marker for breeding. Methods: The *jd30* mutant was phenotypically characterized and compared with the wild type (WT). Genetic analysis was performed using F_2_ populations. Map-based cloning was conducted with simple sequence repeat (SSR) and insertion/deletion (InDel) markers. Candidate genes were annotated and sequenced. Allelism tests were performed by crossing *jd30* with other *lg1* mutants. A Cleaved Amplified Polymorphic Sequence (CAPS) marker was developed based on the causal mutation. Results: The *jd30* mutant showed complete ligule loss from the V2 stage onward and had a significantly smaller leaf angle than the wild type. The phenotype is controlled by a single recessive nuclear gene, mapped to a 123-kb interval on chromosome 2 containing four candidate genes. A single-base cytosine (C) deletion at nucleotide 522 of *ZmLG1* caused a frameshift and premature stop codon. Two other *lg1* mutants, *20292* and *29163*, carried distinct mutations—a C insertion and multiple base substitutions, respectively. Allelism tests confirmed *jd30* as a novel allele of *ZmLG1*. A co-dominant CAPS marker, *M5*, was developed to distinguish wild-type and mutant alleles. Conclusions: This study identifies a novel loss-of-function allele of *ZmLG1*, designated *ZmLG1-1*, and the *M5* marker, providing valuable genetic resources for modifying leaf architecture and improving maize plant compactness.

## 1. Introduction

As a major global crop for food, feed, and bioenergy, maize plays a vital role in ensuring food security [1]. With growing populations and limited arable land, increasing maize yield per unit area has become an urgent priority. Increasing planting density is one of the most effective approaches to improve population yield; however, excessive competition for light resources within dense populations often restricts productivity. Therefore, breeding maize varieties with optimized plant architecture and enhanced density tolerance has become an important goal of modern maize improvement. An ideal plant type characterized by a compact canopy structure can improve light interception and utilization efficiency under high-density conditions. Leaf angle, a key determinant of maize compactness, directly influences the canopy’s light interception and utilization efficiency [2]. Generally, varieties with smaller leaf angles exhibit a more compact plant type [3]. These varieties allow greater light penetration through the upper canopy, maintaining adequate illumination for the middle and lower leaves and improving overall light energy utilization [4]. Recent studies have further demonstrated that genetic regulation of leaf angle and canopy architecture plays an important role in improving maize adaptation to high-density planting, highlighting leaf angle as a key target for density-tolerant breeding [5,6]. Therefore, understanding the genetic basis of leaf angle regulation is crucial for optimizing maize architecture and developing high-density-adapted cultivars.

At the anatomical level, leaf angle is largely determined by differential cell proliferation and expansion within the ligular region. The size and number of parenchyma and sclerenchyma cells in the adaxial (upper) and abaxial (lower) surfaces of the ligular region are among the factors that influence leaf angle [7,8], thereby influencing canopy structure and yield potential [9,10]. Therefore, elucidating the molecular mechanisms underlying ligular region development provides an important foundation for understanding leaf angle regulation and improving maize plant architecture.

The formation and function of the ligular region are controlled by a complex genetic network involving multiple developmental regulators. Based on their effects on ligular region development and leaf architecture, the key regulatory genes can be classified into three categories: The first category, characterized by complete deletion of the ligule region and auricles, is represented by *ZmLG1*, which encodes an SBP-domain transcription factor [11]. Functional deletion of *ZmLG1* disrupts cell polarity differentiation in the ligular region, preventing ligule formation [12]. Its homologs are conserved in rice (*OsLG1*) [13,14], wheat (*TaCP4*) [13], and barley (*HvLG1*) [15]. The second category is marked by partial deletion of the auricular region, including *ZmLG2*, which encodes a bZIP protein that regulates *LG1* through a non-cell-autonomous pathway [16,17]; *ELI-A*, containing an RNaseH domain that maintains the sheath-blade boundary [18]; and *CUL4*, encoding a BTB-Ankyrin protein involved in petiole, leaf axil, and axillary meristem development [19]. The third category involves ectopic expression in the auricular region, where mutations in KNOX family genes (*ZmLG3* and *ZmLG4*) cause the leaf midrib to transform into a sheath-like structure, disrupting organ boundaries [20,21]. Together, these studies demonstrate that ligular region development is genetically complex, with *ZmLG1* serving as a central regulator of ligule formation and leaf angle control.

The maize *LG1* gene serves as a master regulator of ligule morphogenesis and defines the canonical liguleless phenotype. As a transcription factor, *ZmLG1* directly controls the proliferation and polarity establishment of adaxial-abaxial parenchyma cells in the collar region by activating auxin transport genes such as *ZmPIN1*, thereby governing ligule development [5]. In addition to these protein-coding genes, natural variation in cis-regulatory elements, such as the teosinte-derived *UPA2* locus, can also quantitatively modulate leaf angle by fine-tuning the expression of downstream genes involved in the *LG1* regulatory pathway [22]. In contrast to such quantitative regulators, loss-of-function mutations in the *ZmLG1* coding sequence cause a complete loss of ligule and auricle development. Loss-of-function mutants (e.g., the classical *lg1-R* allele) exhibit erect leaves with a significantly reduced leaf angle due to the absence of the ligule [23], which significantly improves canopy light penetration and density tolerance [10,11]. Although *ZmLG1* is recognized as a key regulatory gene, natural allelic variation in maize remains limited. Only a few alleles (such as *lg1-R*) have been identified, restricting their use in breeding programs. Moreover, the absence of efficient and specific molecular markers has further hindered its practical application in developing density-tolerant maize plant architecture.

In this study, we used the maize liguleless mutant *jd30* to clone and characterize the gene underlying the liguleless phenotype. We hypothesized that the liguleless phenotype in *jd30* is controlled by a single recessive locus, and that the causal mutation resides in a known gene involved in ligule development, particularly *ZmLG1*. The specific objectives of this study were: (1) to map and clone the gene responsible for the liguleless trait in *jd30* using F_2_ populations and map-based cloning strategies; (2) to determine whether *jd30* carries a novel allele of *ZmLG1* through candidate gene sequencing and allelism tests with other *lg1* mutants; and (3) to develop a functional codominant marker based on the causal SNP for marker-assisted selection of this allele in maize breeding programs. Through phenotypic characterization, genetic analysis, gene mapping, and allelism testing, we identified *ZmLG1* as the gene underlying the mutant phenotype and regulating leaf angle. Based on functional SNP variations in *jd30* relative to the WT *ZmLG1* allele, a codominant marker, *M5*, was developed. This allele provides a valuable genetic resource for modifying leaf architecture and improving maize plant compactness, and the *M5* marker offers an efficient tool for breeding compact maize plant types.

## 2. Materials and Methods

### 2.1. Experimental Materials

The maize liguleless mutant *jd30*, which originated from the parent inbred line JD30 of the cultivar Shannong 206, demonstrated genetic stability through successive generations of self-pollination. For gene mapping, we crossed *jd30* with two inbred lines, KLx9801 and B73 (both with normal ligule structures), to generate two separate F_2_ populations. The *jd30* × KLx9801 F_2_ population was planted in a field of approximately 736 m^2^ with a row spacing of 60 cm and plant spacing of 15 cm, containing approximately 8200 plants. The *jd30* × B73 F_2_ population was planted in a field of approximately 670 m^2^ under the same spacing conditions, containing approximately 7500 plants. KLx9801 is a near-isogenic line of Lx9801 carrying the resistance gene *qMrdd1* to maize rough dwarf disease, designated with the prefix “K” for resistance. The allelism test included two additional *LG1* mutants, *20292* and *29163*, which have distinct genetic backgrounds and are spontaneous mutants identified by our laboratory. The *LG1* knockout mutant *lg1-cr* was kindly provided by Dr. Chuanxiao Xie’s group at the Chinese Academy of Agricultural Sciences (CAAS). All plants were grown in the experimental fields of Shandong Agricultural University in Tai’an and Hainan.

### 2.2. Leaf Angle Measurement

The *jd30* mutant and its sister line JD29, both derived from the progeny of 1145 and CL313, were planted for leaf angle measurement. Both genotypes were planted in the experimental fields with 4 m row length, 25 cm plant spacing, and 60 cm row spacing. Ten days after pollination, 10 mutant and 10 WT plants were randomly selected to measure leaf angle, with three leaves per plant (the ear leaf, the first leaf above the ear, and the first leaf below the ear) measured and averaged for statistical analysis. Leaf angle was measured manually using a protractor placed at the collar region between the leaf blade and the stem. Differences between datasets were evaluated using a *t*-test. Statistical analyses were performed using Excel, while data processing and graphing were conducted in GraphPad Prism 9.0.

### 2.3. Construction of Segregating Populations and Genetic Analysis

To construct the segregating populations for genetic analysis, we crossed the *jd30* mutant with two wild-type inbred lines, KLx9801 and B73, to generate F_1_ hybrids. The F_1_ plants were then self-pollinated to produce two independent F_2_ populations. For each F_2_ population, the number of plants exhibiting the ligule-present (wild-type) and liguleless (mutant) phenotypes was recorded at the V5–V6 stage, when the ligule phenotype becomes clearly distinguishable. The observed segregation ratios were then compared with the expected Mendelian ratio of 3:1 for a single recessive nuclear gene using a chi-square (χ^2^) goodness-of-fit test.

### 2.4. Mapping of the Liguleless Gene

Genomic DNA was extracted using the sodium dodecyl sulfate method [24]. Initially, 208 pairs of Simple Sequence Repeat (SSR) markers evenly distributed across the ten chromosomes were used to screen for polymorphic loci between parents. Linkage analysis was performed using Bulked Segregant Analysis [25]. Preliminary mapping was conducted using 237 F_2_ liguleless plants derived from the *jd30* × KLx9801 cross. Within the identified mapping interval, new InDel markers were developed based on the B73 RefGen_v4 reference genome sequence. Fine mapping was performed using 2047 F_2_ liguleless plants from the *jd30* × KLx9801 cross and 1865 from the *jd30* × B73 cross. All primer pairs used in this study were designed with Oligo 7 software, and detailed primer sequences are listed in Table S1.

### 2.5. Identification of Candidate Genes and Allelism Tests

The B73 reference genome version 4 (http://www.maizegdb.org/, accessed on 10 July 2023) was used as the reference for candidate gene annotation. Functional annotation was performed to determine the potential biological functions, domain features, and known homologs of the candidate genes. *Zm00001eb067740* (*ZmLG1*) was previously reported to regulate ligule development in maize [11]. To test whether the liguleless phenotype of mutant *jd30* resulted from a loss of function in *ZmLG1*, multiple primer pairs (Table S1) were designed based on the *ZmLG1* sequence from the B73 inbred line. The sequencing data were assembled and aligned using SnapGene software 4.2.4 to detect sequence variations between the WT and mutant alleles.

*jd30* was crossed with two other *LG1* mutants (*20292* and *29163*) with different genetic backgrounds, as well as the knockout mutant *lg1-cr*. The ligule and auricle phenotypes of the resulting F_1_ plants were then examined.

### 2.6. RNA Extraction and Quantitative Real-Time PCR (RT-qPCR)

The ligular region of the third-leaf from mutant *jd30* and WT plants with identical genetic backgrounds was sampled at three developmental stages: S1 (9 days after sowing, ligule 0.2 cm above the stem base), S2 (11 days after sowing, ligule base at half to two-thirds the height of the second leaf ligule), and S3 (13 days after sowing, ligule 1 cm above the second leaf ligule). Approximately 0.5 cm segments of the sampled ligular regions were collected. For each group, ten uniformly developed plants were selected, and three biological replicates were prepared. Total RNA was extracted using a plant RNA extraction kit (Kangwei Century, Beijing, China), and cDNA was synthesized using a reverse transcription kit (Móna Biotechnology, Beijing, China). RNA quality was assessed by 1% agarose gel electrophoresis to verify integrity, and by BioDrop spectrophotometry (BioDrop, Cambridge, UK) to determine concentration and purity (OD260/280 ratio). RT-qPCR was conducted using PerfectStart™ Green qPCR SuperMix (Beijing TransGenBiotech Co., Ltd., Beijing, China) on a QuantStudio™ 6 Flex Real-Time PCR System (Applied Biosystems, Thermo Fisher Scientific, Singapore) with *GAPDH* (*Zm00001d049641*) as the reference gene, whose expression stability in maize has been previously validated [26]. Raw data were acquired using QuantStudio™ Real-Time PCR Software v1.7.1 (Applied Biosystems, Thermo Fisher Scientific). Relative expression levels of the four candidate genes in the ligule tissues were calculated using the 2^−ΔΔCt^ method (where Ct denotes the cycle threshold) [27]. The amplicon lengths were as follows: *Zm00001eb067710*, 173 bp; *Zm00001eb067720*, 157 bp; *Zm00001eb067730*, 175 bp; *ZmLG1* (*Zm00001eb067740*), 540 bp; and *GAPDH*, 362 bp. Sequences of all qPCR primers are listed in Table S1.

### 2.7. Development and Validation of CAPS Markers

Sequence differences between mutant *jd30* and B73 reference genome were used to identify SNPs underlying the recessive loss-of-function mutation. CAPS markers flanking the candidate site were designed using dCAPS Finder 2.0 (http://helix.wustl.edu/dcaps/dcaps.html, accessed on 26 March 2024). CAPS marker *M5* was used to genotype F_2_ segregants for the presence or absence of the ligule.

## 3. Results

### 3.1. Phenotypic Identification of jd30

**Figure 1:**
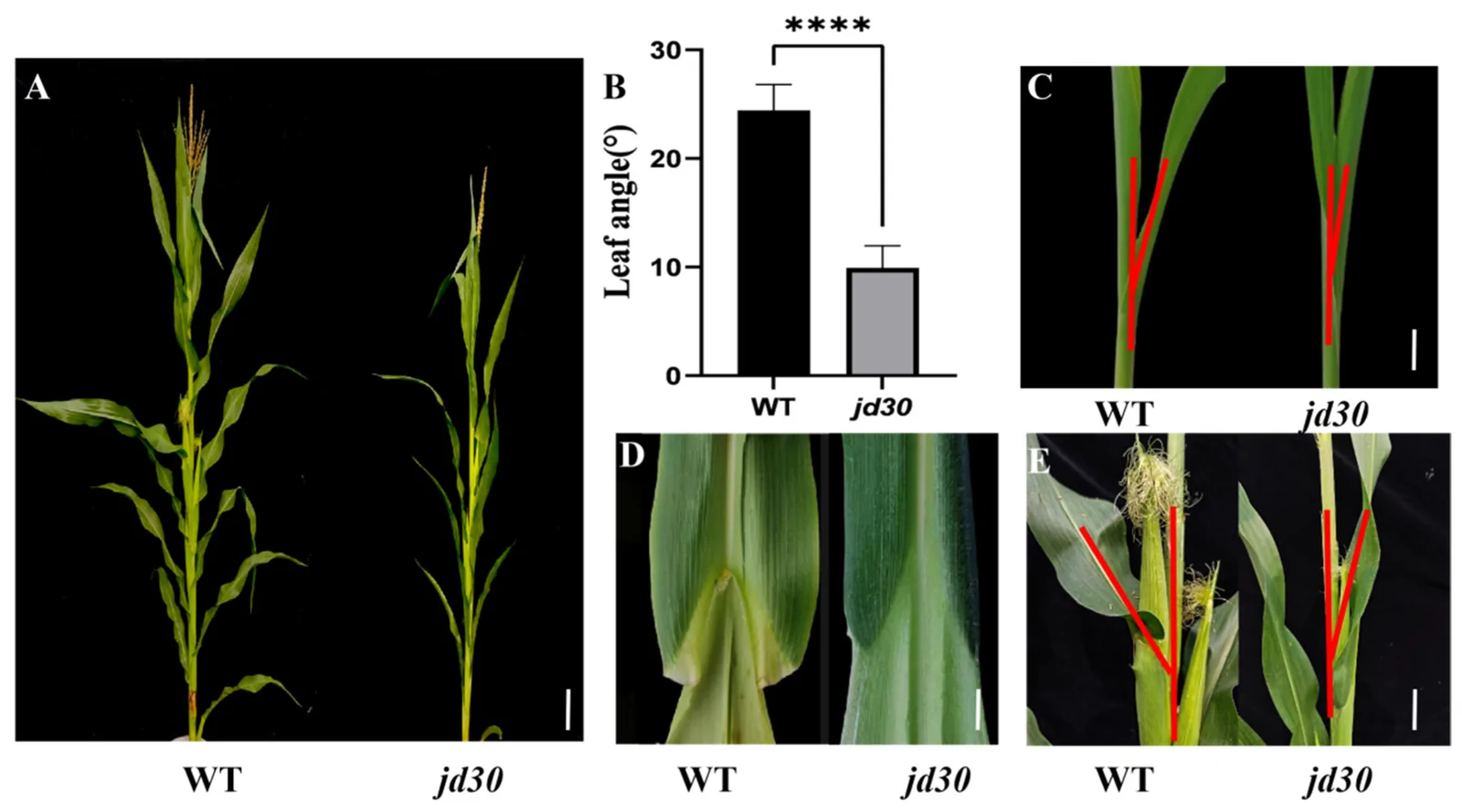
Phenotypic comparison between WT and the *jd30* mutant. (**A**) Phenotypic characterization of WT and *jd30* mutant at R1 stage; scale bar = 12 cm. (**B**) Ear leaf angle quantified from 10 plants per genotype. Bars represent means ± SD. ****, *p* < 0.0001 (two-tailed t-test). (**C**) The first leaf above the upper ear of WT and *jd30* mutant at V2 stage; scale bar = 2 cm. (**D**) The first leaf above the upper ear of WT and *jd30* mutant at R1 stage; scale bar = 6 cm. (**E**) Leaf angle of the first leaf above the upper ear measured at R1 stage; scale bar = 5 cm. Red lines in panels (**C**,**E**) mark the positions for leaf angle measurement.

To determine whether the ligule deficiency affected leaf angle, we measured the angle of the ear leaf at 10 days after pollination in both *jd30* and WT. At R1 stage, the *jd30* mutant exhibited a compact plant architecture with upright leaves compared with WT (Figure 1A). The results showed that *jd30* had a significantly smaller ear leaf angle than WT (Figure 1B). The liguleless phenotype first became visible at the V2 stage and persisted through all subsequent leaves (Figure 1C,D). Throughout development, the mutant displayed an increasingly compact growth habit, characterized by narrow, upright leaves (Figure 1E).

### 3.2. Genetic Analysis and Map-Based Cloning of the Liguleless Locus

All F_1_ plants from crosses between *jd30* and the inbred lines KLx9801 and B73 exhibited normal ligule and auricle development, indicating that the liguleless phenotype is recessive. Genetic analysis of F_2_ populations revealed a segregation ratio of 3:1 for normal to mutant phenotypes (χ^2^_c_ = 3.07 < χ^2^_0.05,1_= 3.84) (Table S2). This indicates that the liguleless trait is controlled by a single recessive nuclear gene.

**Figure 2:**
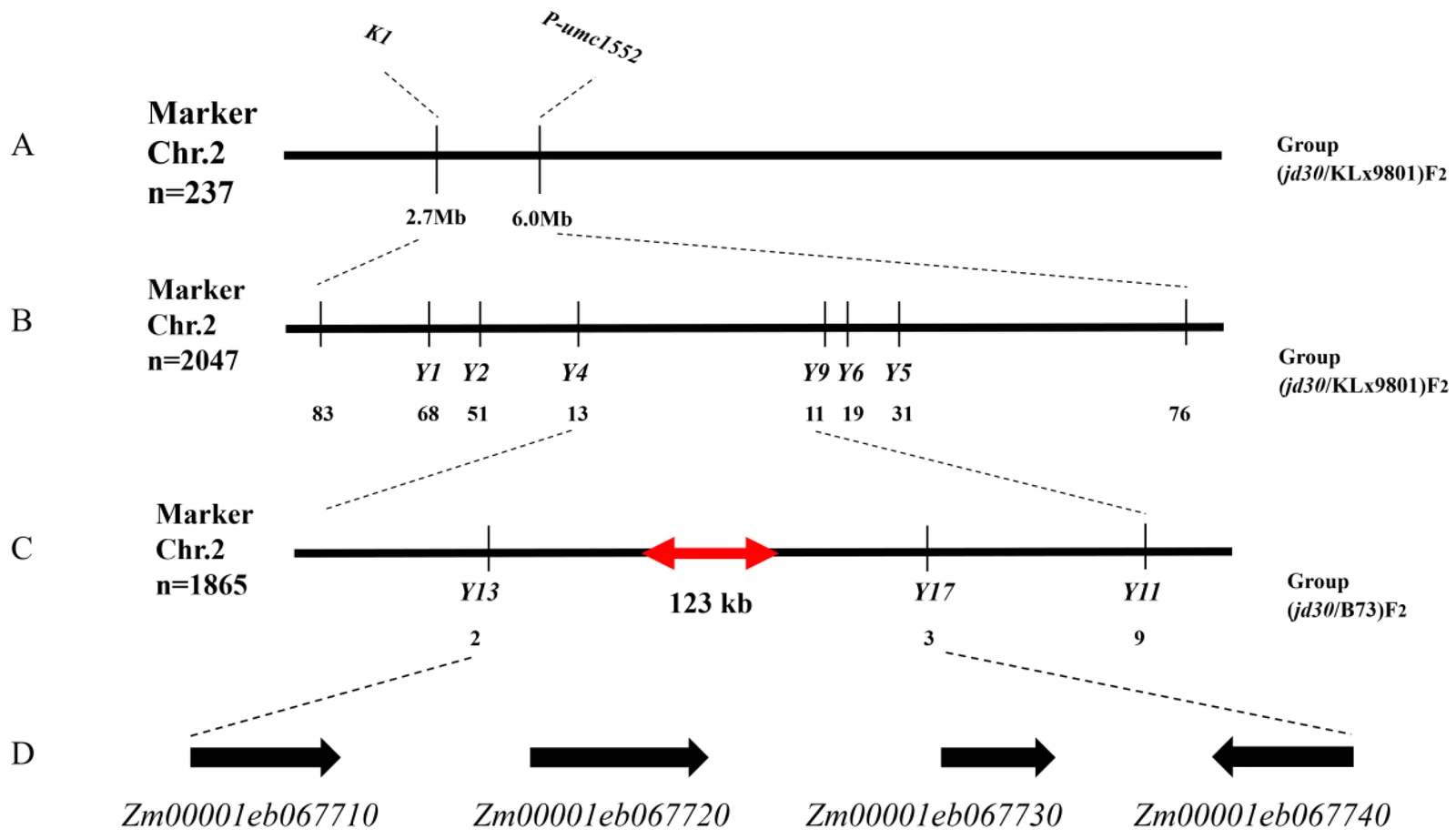
Fine mapping of the *ZmLG1-1* gene. (**A**) The *ZmLG1-1* locus was initially mapped between markers *K1* and *p-umc1552* on the short arm of maize chromosome 2 by using 237 F_2_ liguleless plants from the *jd30* × KLx9801 cross. (**B**) The region was narrowed to the interval between markers *Y9* and *Y4* using *2047* F_2_ mutants. (**C**) Further mapping localized the gene within a 123 kb interval between markers *Y13* and *Y17* using 1865 F_2_ mutants. (**D**) Four candidate genes were identified within the 123 Kb region. Red double-headed arrows in panel (**C**) delimit the 123-kb target mapping interval. Black arrows in panel (**D**) indicate candidate genes, with arrowheads pointing to their transcription direction.

A total of 32 polymorphic markers were identified. These 32 polymorphic markers were used to genotype both parents and the two DNA bulks. The genotypes of the normal and mutant DNA bulks matched those of the parental lines KLx9801 and *jd30*, respectively, suggesting linkage between these polymorphic markers and the target gene. Linkage analysis of the 237 liguleless plants confirmed that the SSR marker *p-umc1552*, located on chromosome 2, was linked to the target gene (Figure 2A). Subsequently, 48 SSR markers on chromosome 2 were designed based on the B73 reference genome (version 4), and the polymorphic marker *K1* was selected for subsequent analysis. Linkage analysis using the same 237 F_2_ liguleless plants confirmed the linkage marker and localized the target gene on chromosome 2 between *p-umc1552* and *K1*, with genetic distances of 2.1 cM and 2.2 cM, respectively.

The mapping interval was further narrowed to a region within the markers *Y9* and *Y4* using newly developed markers (*Y1*, *Y2*, *Y4*, *Y5*, *Y6*, *Y9*, etc.). To further refine this interval, 17 InDel marker pairs potentially linked to the liguleless mutation were developed between *Y9* and *Y4*. Fine mapping was performed using 2047 liguleless F_2_ individuals from the *jd30* × KLx9801 cross (Figure 2B) and 1865 liguleless F_2_ individuals from the *jd30* × B73 cross. The target gene was subsequently delimited to a 123 kb region flanked by markers *Y13* and *Y17* (Figure 2C). This interval encompassed four candidate genes: *Zm00001eb067710*, *Zm00001eb067720*, *Zm00001eb067730*, and *Zm00001eb067740* (Figure 2D).

### 3.3. Identification of ZmLG1-1 as a Novel Allele of ZmLG1

**Figure 3:**
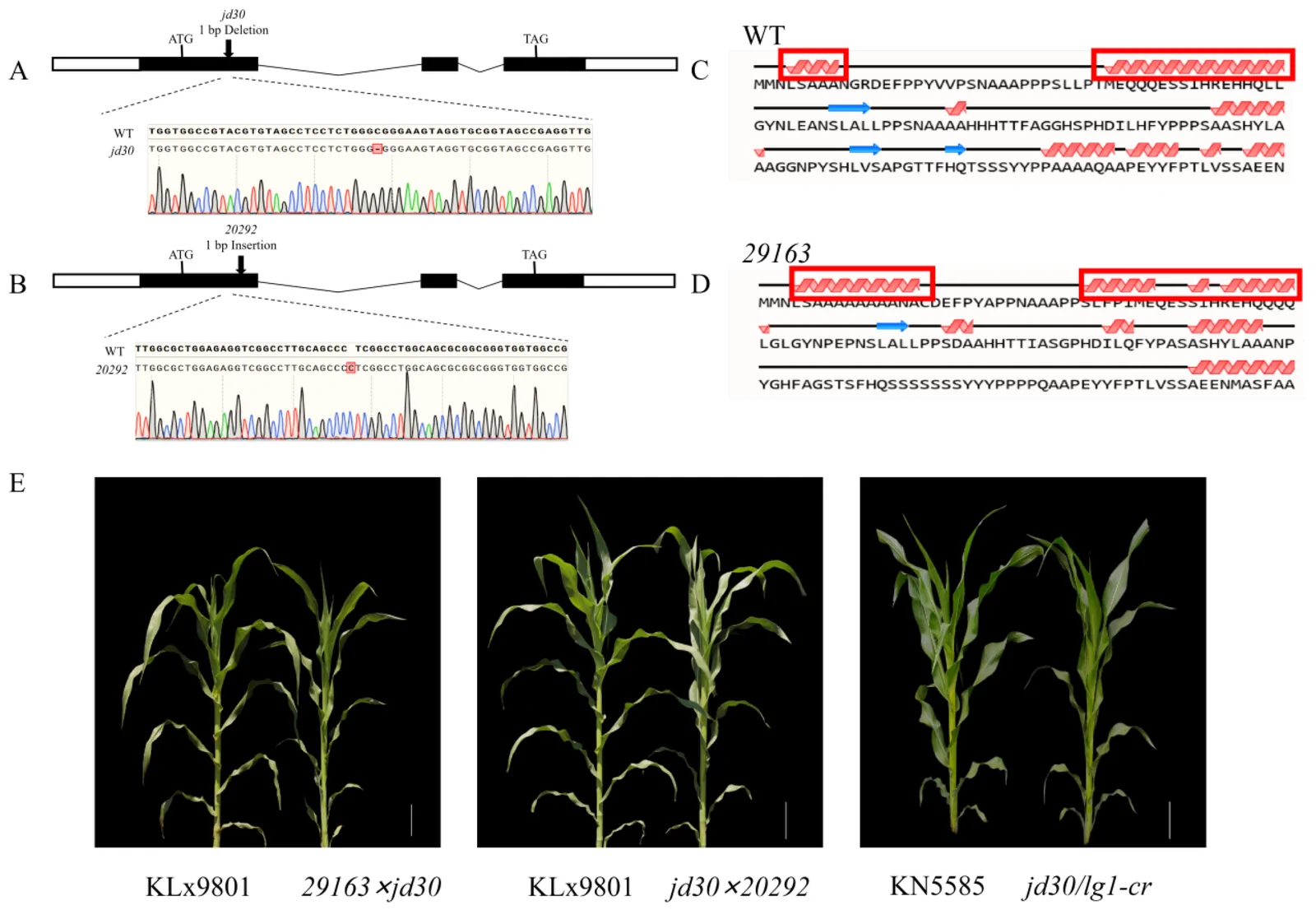
Mutation sites of *ZmLG1-1* and allelism tests. (**A**) Variant site of mutant *jd30*. (**B**) Variant site of mutant *20292*. (**C**,**D**) Predicted secondary structure of the protein encoded by *ZmLG1-1*. (**E**) F_1_ phenotypes of crosses between *29163* × *jd30*, *jd30* × *20292*, and *jd30* × *lg1-cr*; bar = 25 cm. Red boxes in panels (**C**,**D**) highlight regions with altered secondary-structure features between wild-type and the *29163* mutant.

Among the four candidate genes within the mapping interval, *Zm00001eb067740* corresponds to the previously reported *ZmLG1* gene, which is known to control the liguleless trait. Sequencing analysis revealed a deletion at the 522nd nucleotide in mutant *jd30*, resulting in a frameshift mutation and premature termination of translation, likely producing a non-functional protein and causing the liguleless phenotype (Figure 3A). The mutation identified in *jd30* is distinct from those in other known *ZmLG1* alleles. Therefore, we conclude that the gene responsible for the mutant trait in *jd30* is a novel allele of *ZmLG1*, designated *ZmLG1-1*.

To further verify that *ZmLG1-1* is an allele of *ZmLG1*, we sequenced the full-length *ZmLG1* gene from two other liguleless mutants, *20292* and *29163*. Sequencing revealed that *20292* carries a single (C) insertion at the 576 bp position of the coding region, resulting in premature termination of translation (Figure 3B). In mutant *29163*, multiple nucleotide substitutions were identified in the coding region, leading to amino acid changes that may affect the predicted protein secondary structure (Figure 3D).

Furthermore, allelism tests were conducted by crossing *jd30* with *20292*, *29163*, and the *lg1-cr* knockout mutant. All F_1_ progeny from these crosses exhibited a uniform liguleless phenotype (Figure 3E). This confirmed that *jd30* is allelic to *ZmLG1*, consistent with our designation of *ZmLG1-1*.

### 3.4. Candidate Gene ZmLG1 Expression Patterns

**Figure 4:**
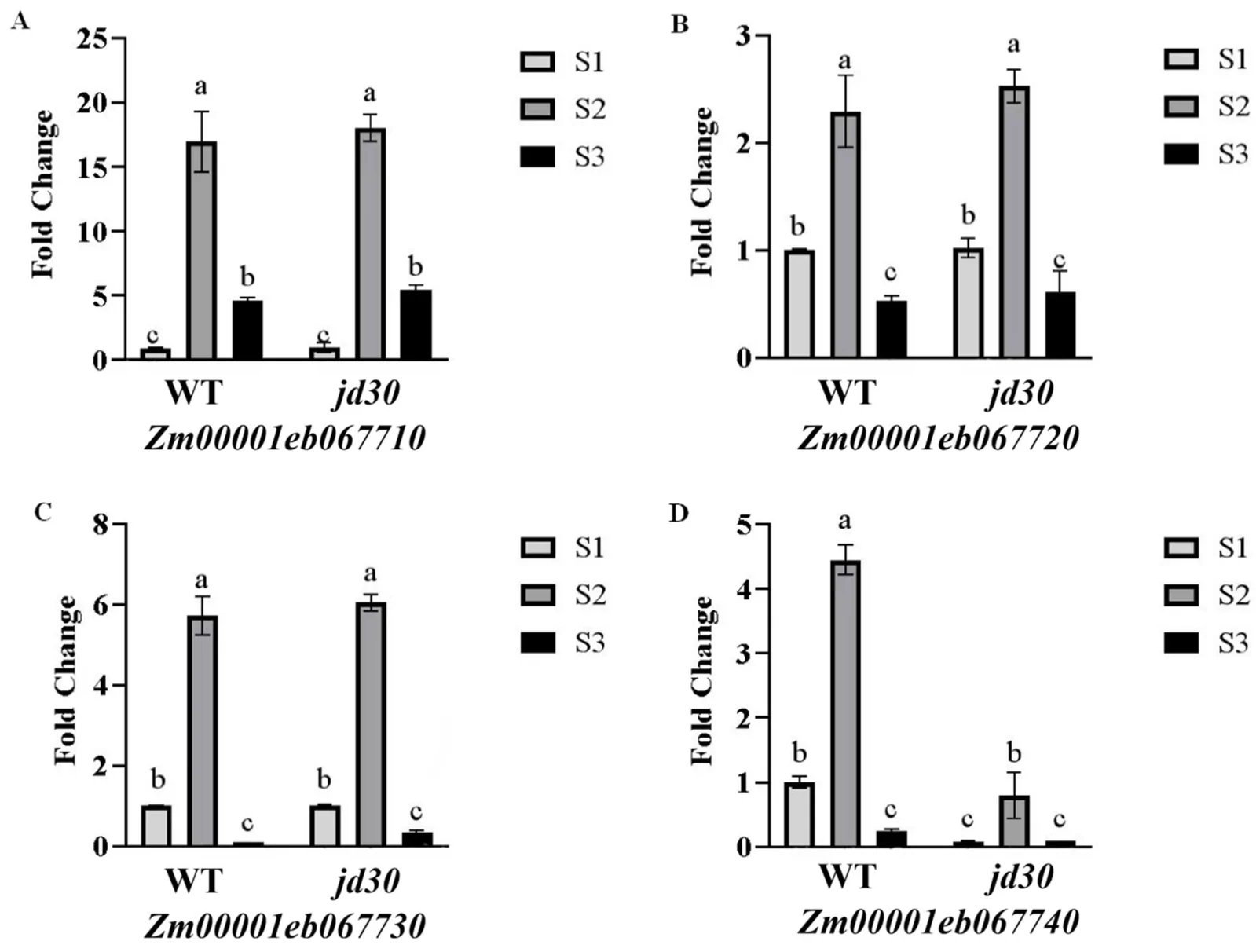
Expression patterns of candidate genes during ligule development. (**A**–**D**) Relative expression levels of *Zm00001eb067710*, *Zm00001eb067720*, *Zm00001eb067730*, and *Zm00001eb067740* (*ZmLG1*) in the ligular region of *jd30* mutant and WT plants at three developmental stages (S1–S3). Expression was normalized to the internal control *GAPDH*. Bars represent means ± SD of three biological replicates. Different lowercase letters (a, b, c) above the bars indicate significant differences between genotypes at each stage (two-tailed *t*-test, *p* < 0.05).

To further identify the target gene, we analyzed the expression levels of the four candidate genes in the ligular region of mutant *jd30* and the WT across three developmental stages (S1, S2, and S3) using RT-qPCR. The expression of *Zm00001eb067710*, *Zm00001eb067720*, and *Zm00001eb067730* showed no significant differences between mutant and WT at any of the stages tested (Figure 4A–C). The results showed that the expression of *Zm00001eb067740* was significantly lower in the mutant than in WT at each of the three stages (Figure 4D). This candidate gene corresponds to *ZmLG1*, which regulates ligule development and leaf angle in maize. The reduced expression of *ZmLG1* in the mutant may thus underlie its abnormal leaf angle and altered plant architecture.

### 3.5. Development and Application of a Functional Marker for the Maize ZmLG1-1 Allele

**Figure 5:**
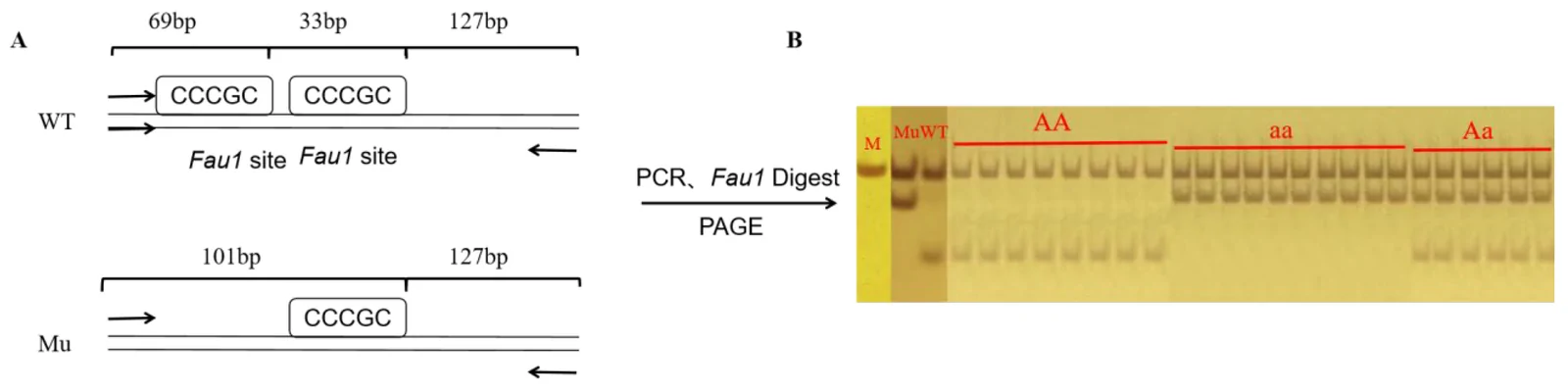
Genotyping of the CAPS marker *M5* and F_2_ population (*jd30* × B73) by restriction enzyme digestion. (**A**) FauI restriction site: WT is cleaved into three fragments, while the mutant (Mu) is cleaved into two fragments by FauI. (**B**) Polyacrylamide gel electrophoresis (PAGE) image of the F_2_ population after digestion. M: DNA molecular weight marker; WT, homozygous wild-type (AA), 127 bp + 69 bp; Mu, homozygous mutant (aa), 127 bp + 101 bp; Heterozygote (Aa), 127 bp + 101 bp + 69 bp. Black arrows represent PCR primer binding sites. Boxes indicate Fau I restriction-enzyme recognition sites.

A co-dominant CAPS marker, *M5*, was developed based on a SNP at position 522 in the coding sequence (CDS) of *ZmLG1-1*, which differentiates WT from the *jd30* mutant. This single-base deletion results in the loss of a FauI restriction site. PCR amplification with specific primers produced a 229 bp product containing two FauI (CCCGC) restriction sites (Figure 5A). Digestion of the PCR product with FauI yielded the following banding patterns: liguleless homozygotes produced 101 bp and 127 bp bands; ligule-present homozygotes produced 127 bp and 69 bp bands (with the 33-bp band undetectable due to its small size); and heterozygotes showed 127 bp, 101 bp, and 69 bp bands (Figure 5B). Collectively, these results demonstrate that the *M5* marker can effectively distinguish ligule-present and liguleless genotypes.

## 4. Discussion

Leaf angle is regulated by multiple genes. Genome-wide association studies (GWAS) results show that variations at the liguleless genes have contributed to more upright leaves [6]. *ZmLG1* belongs to the SBP transcription factor family and contains a conserved SBP domain of 80 amino acids [28]. Sequencing identified a (C) deletion at the 522 bp position in the *ZmLG1-1* coding region (Figure 3A), resulting in premature termination of translation. This mutation, together with other known *ZmLG1* alleles including the (C) insertion at position 576 bp in *20292*, multiple base substitutions in *29163*, and the null allele *lg1-R*, leads to a common phenotypic outcome: loss of ligule formation [12]. The frameshift mutations in *jd30* and *20292* flank a 54 bp interval within the SBP domain, suggesting that this region is critical for *ZmLG1* function. This is further supported by the loss-of-function phenotype of *29163*, an independent mutant with multiple substitutions affecting the C-terminal portion of the SBP domain [11]. Collectively, these data highlight the functional importance of this region.

Compact plant architecture is essential for improving yield under high-density planting conditions. The *jd30* mutant displays a reduced leaf angle, which enhances canopy light penetration. The trait is consistent with the known role of LG1 in regulating leaf angle [29]. The effects of *jd30* are consistent with the involvement of *ZmLG1* in multiple regulatory pathways, including transcriptional regulation by *ZmIDD14* and *ZmTCP23*, as well as phyB-mediated light signaling [30]. This highlights *ZmLG1-1* as a valuable resource for breeding maize lines with compact plant architecture.

Mechanistically, the presence of the ligule and auricle structures at the blade-sheath boundary physically pushes the leaf blade outward, thereby increasing the leaf angle. Conversely, loss of the ligule eliminates this pushing force, allowing the leaf to adopt a more upright orientation [12,30]. At the cellular level, *LG1* functions by establishing adaxial-abaxial polarity in the ligular region through the activation of auxin transport genes such as *ZmPIN1*, which directs cell proliferation and expansion patterns that ultimately determine leaf angle [5]. The frameshift mutation in *ZmLG1-1* disrupts this regulatory cascade, resulting in the absence of ligule formation and consequently reducing leaf angle. In addition to *ZmLG1*, other genes and pathways have been reported to regulate leaf angle, as partially described above and in other studies [22,30], with *ZmLG1* serving as a central node in a complex regulatory network. Compared with other leaf traits such as leaf area or leaf length, leaf angle has a more direct impact on canopy light penetration under high-density planting conditions. While leaf area and leaf length primarily affect photosynthetic surface area, leaf angle determines the spatial distribution of leaves within the canopy, influencing light interception at different layers [4,6]. Thus, leaf angle is considered a primary target for high-density breeding, with other leaf traits serving as secondary modifiers of plant architecture [22,31].

Beyond these direct effects, *jd30* also displays variation among its progeny. In the F_2_ population derived from a cross with inbred line B73, leaf angles exhibited a continuous distribution, categorized into three types: spreading (25–30°), intermediate (11–14°), and compact (4–7°). The segregation of spreading, intermediate, and compact leaf angle types indicates that *ZmLG1-1* has a major effect on leaf angle determination, while additional genetic factors from both parental backgrounds contribute to quantitative modification of this trait. Similar genetic background effects have been reported for leaf angle regulation by *ZmLG1* and *UPA2* [22,31]. The intermediate-type plants may represent a balanced canopy architecture between spreading and compact types [32], providing potential genetic resources for optimizing maize plant architecture. Whether these architectures confer advantages under high-density planting conditions requires further evaluation.

However, efficient utilization of these materials in breeding programs requires reliable molecular markers. Most currently available markers for leaf angle are linked markers rather than functional markers, meaning they co-segregate with the trait but do not directly target the causal polymorphism, limiting their accuracy and transferability across genetic backgrounds. In contrast, the *M5* marker directly targets the key SNP responsible for protein loss of function. This co-dominant marker shows high accuracy in co-segregating with the phenotype and can be used for large-scale screening of maize germplasm to identify materials carrying natural *ZmLG1* loss-of-function alleles, thereby broadening the genetic base for breeding. Importantly, the newly identified allele was derived from a breeding-relevant genetic background, rather than from a classical mutant source with unknown breeding value. This genetic background may facilitate its utilization in maize improvement.

It is worth noting that classical *lg1* mutants are predominantly loss-of-function alleles that result in the complete absence of ligule and auricle structures, leading to pronounced phenotypic alterations, including altered canopy patterns and dramatically decreased photosynthetically active radiation (PAR) interception under high-density conditions [11]. Although these mutants have been instrumental in elucidating the genetic regulation of ligule development, their drastic morphological effects may limit their direct application in breeding programs. By contrast, recent studies have shown that certain naturally occurring allelic variants at the *LG1* locus can fine-tune leaf angle through moderate effects on ligule development, without completely eliminating these structures [33]. This type of allelic variation, rather than complete loss of gene function, is more favorable for optimizing plant architecture in high-density planting systems [22]. The *ZmLG1-1* allele identified in this study, which originates from the elite inbred line JD30 with compact plant type and reduced leaf angle, represents a more practical genetic resource for maize breeding compared with classical null mutants. In addition, the paternal line belongs to a heterotic group distinct from that of the maternal line, which may further enhance its breeding potential through heterosis.

Future research will focus on elucidating the downstream network of *ZmLG1-1* in regulating ligule development and clarifying how environmental signals modulate its function. Given that phyB physically interacts with *ZmLG1-1* to regulate plant architecture and density tolerance [34], it will be important to investigate whether the *ZmLG1-1* allele affects this phyB-*LG1* interaction or whether the density-dependent performance of intermediate-type plants is mediated through this light-signaling pathway. *M5*-based marker-assisted backcrossing will accelerate the introgression of *ZmLG1-1* into inbred lines, allowing yield evaluation at different planting densities.

## 5. Conclusions

In this study, we cloned and characterized a new maize liguleless allele, *ZmLG1-1*, which results from a single-base deletion (C) at position 522 in the coding region of *ZmLG1*, causing premature termination of SBP protein translation. Sequencing of two additional liguleless mutants revealed that *20292* carries a (C) insertion at position 576 bp, also causing premature translational termination, while *29163* contains multiple base substitutions that alter its amino acid sequence and predicted secondary structure. Based on this functional SNP, we developed a co-dominant CAPS marker, *M5*, which enables precise genotyping and provides an efficient tool for marker-assisted selection. This marker and the newly identified allele provide valuable genetic resources for modifying leaf architecture and improving maize plant compactness.

## Data Availability

The original contributions presented in this study are included in the article/Supplementary Material. Further inquiries can be directed to the corresponding author(s).

## Supplementary Materials

The following supporting information can be downloaded at https://www.mdpi.com/article/10.3390/genes17091075/s1, Table S1: Primers for partial gene mapping, cloning, and expression analysis; Table S2: Segregation analysis of mutant phenotype in F_2_ populations of maize mutant *jd30*.

## Abbreviations

The following abbreviations are used in this manuscript:

CAPS	Cleaved Amplified Polymorphic Sequence
CDS	coding sequence
GWAS	genome-wide association study
InDel	insertion/deletion
LG1	Liguleless 1
RT-qPCR	quantitative real-time PCR
SNP	single-nucleotide polymorphism
SSR	simple sequence repeat
WT	wild type
PAR	photosynthetically active radiation
