# Supplementary material for: A Novel ZmLG1 Allele as a Genetic Resource for Breeding Compact Plant Architecture in Maize
Source: Genes (Basel). 2026 Sep 6;17(9):1075. doi: 10.3390/genes17091075 (PMC13606650; doi:10.3390/genes17091075)
Supplement: Supplementary file 1 [file genes-17-01075-s001.zip › genes-4499940-supplementary.pdf]

Table S1. Primers for partial gene mapping, cloning, and expression analysis

Primer Name	Forward Primer (5'→3')	Reverse Primer (5'→3')
	Gene Mapping Primers	
p-umc1552	CTCGATAGCTCTGCTGCTTCCTC	CAACACCAGCCCTACCCAGA
K1	TCTTTCTTTCTCCGCCGAG	GACCCGAATTATTGCACCC
Y1	GTACGCGAGATCTGTGCTTG	GGAGAAAGAAGCTAACTGACCTT
Y2	GGAGAAAGAAGCTAACTGACCT	TCCCCAGCGAAATCAAATGC
Y4	GGTATAATTGTTCAGGCTGGGG	CAACTGCATCAAAACAAGGGG
Y5	GCACATTCGAACAGGTACCC	GGAGACTAGACGATCTGCCC
Y6	AGATGAGACCGGTCCTTTGG	GCCTGTGCCTCTTACCAAGT
Y9	TGGACGTTGGAGATATGCCC	AAATTCACCGAGACCCACCC
Y11	TGCCTCAGGTCATTTCAGGT	CCTTTCCTTTCCCTTGAGCAC
Y13	AGTCGCTGGGATGCATTTTC	AACTGATTCAACTAAGGGTGCT
Y17	GTCCCGTCCATCCATTCCTC	CCCTATACACGTGAACATGCA
	Gene Cloning Primers	
G1	AATCCGTTGGCCAGAGAAGA	GGAGCACTTAAATGGCTAGC
G4	ACTTCGTCTCTTCAGCTGCA	TACTTCACACGTGCATGCTC
G5	CCACAGCCAGCCTAGCATAT	ACTGGGATGTGAACTGACGAG
G9	TACGTACGTACGTCTACCCG	GCAACCACTACTTCTCGATC
G10	GTCCCGTCCATCCATTCCTC	CCCTATACACGTGAACATGCA
	RT-qPCR Primers	
QP7710	GACACATTGGCGGAAGGACGA	ATGCCGCCCATGTTCTTCTCGACA
QP7720	TGTACGCTGCAAAGCTTGATGGC	TCCAGGAGCCTTGACGACTTCA
QP7730	ACAAGTTCGTGCGGCGGATCTA	GTTGTTGAAGTAGGCGTTGTCGA
QP7740	GCGTGGGAAGATGATGAACC	GTACGTGTGCCTCCTCTGG
GAPDH	CCATCACTGCCACACAGAAAAC	AGGAACACGGAAGGACATACCAG
	CAPS Marker	
M5	GAACATGGCCAGCTTCGCCGCC	GAACCTCTGATGCAGTCCACCGGC

Table S2. Segregation analysis of mutant phenotype in F_2 populations of maize mutant *jd30*

Population	Total plants	Normal plants	Mutant plants	Expected ratio	$\chi^2_{0.05}$
(*jd30*/KLx9801) F_2	978	741	237	3:1	0.307
(*jd30*/B73) F_2	1650	1248	402	3:1	0.356
